# Artificial Intelligence and Prostate–Breast Cancer Biology: Recent Advances in Treatments and Perspectives — A Comprehensive Review

**DOI:** 10.1002/hsr2.72117

**Published:** 2026-03-16

**Authors:** Russell B. O. Ouma, Silas M. Ngari, Joshua K. Kibet

**Affiliations:** ^1^ Department of Chemistry Egerton University Njoro Kenya

**Keywords:** breast cancer, mortality rates, nanomedicine, oncogenesis, Prostate cancer, targeted therapies

## Abstract

**Background and Aims:**

The therapeutic limitations of conventional cancer treatment currently deployed are driving the development and creation of new nano‐drugs. Prostate cancer (PCa) is a prevalent clinical problem in older men with a significant global incidence, with 1.5 million cases reported in 2022. Breast cancer (BC) is also a considerable health burden among women worldwide, with reports showing over 67,000 deaths in 2022. Generally, across the world, cancer has posed significant health burden due to limited access to treatment options, high incidence rates, and mortality rates occasioned by lifestyle and genetic factors. This review explores the potential of nanomedicine and artificial intelligence (AI) to overcome these limitations and improve patient outcomes, and provides an insightful overview of the available cancer treatment options, such as radiation therapy, surgery, hormone therapy, chemotherapy, and targeted therapies, focusing on PCa and BC.

**Methods:**

A comprehensive synthesis of recent literature has been conducted, focusing on enormous advances that have been made to understand the biology of various cancers, imaging technologies, and therapeutic approaches. Subsequently, cancer management has evolved rapidly, focusing on the genomic landscape and oncogenesis of localized and metastatic cancers.

**Results:**

The current treatment strategies face therapeutic challenges related to accessibility issues, drug resistance, and side effects. The emergence of nanomedicine and artificial intelligence (AI) models in imaging, therapy, and drug delivery offers solutions related to these challenges. Nanomedicine is a growing research hotspot with an increased number of publications with keywords such as “prodrug,” “cancer nanomedicine,” “cancer immunotherapy,” “artificial intelligence,” and “targeted nanomedicine”.

**Conclusions:**

The nanotechnologies utilized in cancer treatment reduce side effects, enhance treatment efficacy, and improve patient outcomes. Combining nanomedicine with conventional therapies holds promise in revolutionizing cancer treatment and addressing the clinical challenges of current therapies.

## 1 Introduction

Cancer is one of the most acute problems in biomedical science and clinical medicine, not only because of its burden globally but also because of the complexity of its biology and the perpetual development of resistance to therapeutic interventions [1, 2]. Present‐day genetic disease framing of cancer evolved over decades of discovery [3]. By the mid‐20th century, cancer was seen as an uncontrolled growth of cells, and histopathological classification came into focus [4]. The discovery of tumour protein 53 (TP53), retinoblastoma (RB1), and myelocytomatosis (MYC) in the 1970s and 1980s offered a mechanistic understanding of how genetic changes contribute to tumour development, shifting pathology towards molecular causation [5]. Prostate‐specific antigen (PSA) testing, introduced in the late 1980s, transformed prostate cancer (PCa) screening by providing a biomarker‐based method of early detection, proving the significance of molecular markers in cancer management despite shortcomings [6]. By the early 2000s, cancer as a genome disease was established by the sequencing of the human genome and later cancer genome projects, where mutational landscapes vary across tumour types [7]. Another turning point was the 2010s clustered regularly interspaced short palindromic repeats‐Cas9 (CRISPR‐Cas9) revolution, which allowed researchers to explore how individual genes contributed to cancer biology through functional genomics [8]. Whole‐genome CRISPR knockout screens demonstrated resistance to therapies [9], showing how cancer knowledge evolved beyond descriptive pathology to mechanistic genomics and interrogation of vulnerabilities. The three pillars of treatment—surgery, chemotherapy, and radiation—have shortcomings, particularly toxicity and lack of specificity [10]. Subsequently, it prompted targeted therapies and immunotherapies such as tyrosine kinase inhibitors, monoclonal antibodies, checkpoint inhibitors, and chimeric antigen receptor (CAR‐T) cells [11].

Among all cancers, BC and PCa have emerged as the second and third most prevalent types of cancer since 2018, respectively [12]. There are over 1 million deaths globally due to metastatic PCa and BC [13]. Precise diagnostics and screening are recommended to determine an appropriate PCa and BC treatment course. Early detection of these cancers augments the quality of life, overall survival rate, and timely decision‐making [14]. Breast biopsies are a common invasive method used to detect BC potential risks, including bleeding, bruising, infection, and swelling of the biopsy site [15]. Furthermore, urinary tract diagnosis in a non‐invasive (pain‐free) approach can easily detect specific biomarkers, such as microRNAs (miRNAs) that regulate cell metabolism. On the other hand, serum prostate‐specific antigen (PSA) is a pivotal biomarker in PCa diagnosis, showing substantial promise in reducing PCa‐related mortality [16]. However, the diagnosis of both cancers has escalated debate as to whether screening does more harm than good, mainly due to the unintended consequences of overtreatment and excessive diagnosis of low‐risk cancers [17, 18]. Molecular knowledge has been transferred to clinical innovation, including targeted agents like poly (ADP‐ribose) polymerase (PARP) inhibitors in breast cancer, and androgen receptor antagonists used with prostate cancer [19]. However, genetic heterogeneity and adaptive signalling can enable resistance mechanisms, which are a challenge and highlight the need to combine biology, nanotechnology, and AI. The field of nanomedicine originated in the early 2000s as a reaction to the shortcomings of traditional drug delivery [20]. Initial clinical trials of liposomal formulations, including liposomal doxorubicin, showed enhanced pharmacokinetics and lower cardiotoxicity relative to free drug [21]. Similarly, albumin‐conjugated paclitaxel (nab‐paclitaxel) nanoparticles improved delivery and efficacy in breast cancer [22]. These experiments played a critical role in confirming the viability of nanocarriers, and basic surveys of the time identified the promise of nanoparticles in controlled release, tumour targeting, and theranostics.

The treatment and management options of PCa and BC depend on the stage and type of cancer [23]. The common treatments for BC include chemotherapy, radiation therapy, surgery, and so forth [24]. PCa patients have also benefited from treatment options, including active surveillance, surgery, chemotherapy, and radiation therapy, among others [25]. Nevertheless, these treatment options are associated with significant clinical challenges related to drug‐related side effects, including cisplatin‐induced ototoxicity and nephrotoxicity, anthracycline‐induced cardiotoxicity, cardiac and pulmonary injury, and radiation dermatitis [26]. Nanotechnology has come to light in improving cancer treatment through thermal therapy, targeted drug delivery, gene therapy, and diagnostics and imaging. Nanoparticles can be optimized to deliver therapeutic molecules and allow imaging that gives dynamic feedback of drug activity. This twofold capability demonstrates that nanotechnology is laying the groundwork between biology and AI‐driven analytics [27].

Among the most recalcitrant challenges in oncology is therapy resistance. Conventional methods of resistance study were based on cell line modelling and stepwise mechanistic experiments. This landscape changed with the introduction of CRISPR screening, which facilitated the systematic identification of resistance genes. Among these is the study of Liu et al. [9], which employed genome‐wide functional screens to understand new mediators of resistance to targeted therapies. These illustrations provided in the introduction emphasize the translational nature of functional genomics and position AI as a natural companion in the analysis of the volumes of data generated in such screens. In this review, we cover the best diagnosis and management of BC and PCa in the healthcare setting. The objectives of this review paper are threefold: (1) to assess the epidemiological trends and global burden of BC and PCa, (2) to examine the current therapeutic approaches for BC and PCa, and (3) to provide a concise overview of how nanotechnology and AI‐assisted imaging have improved cancer management. Accordingly, the paper offers a concise overview of PCa and BC progression, the synergistic relationship between nanomedicine and conventional cancer treatments, and the prospects of AI‐assisted imaging in improving cancer screening and treatment.

## 2 Methodology

**Figure 1:**
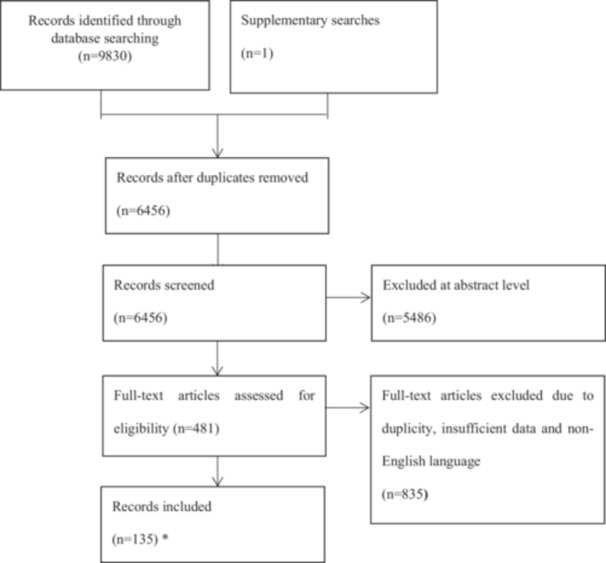
Flowchart methodology used in this review paper.

In this review, we collected reviews and studies concerning treatment options currently deployed in BC and PCa management, published from 2001 to the present in Scopus, Web Science, WHO Global Health Library, Google Scholar, medRxiv, and PubMed databases. The keywords used in the literature search were: “prostate cancer”, “breast cancer”, “chemotherapy”, “radiation therapy”, “radical prostatectomy”, “external beam radiation therapy”, “surgery”, “immunotherapy”, “active surveillance”, “brachytherapy”, “hormone therapy”, “nano drugs”, “artificial intelligence”, and “prodrugs”. Accordingly, terms like “prodrugs”, “artificial intelligence”, and “nano‐drugs” were used to capture current technological and innovative developments in cancer therapy. The selection of scholarly materials from the early 2000s (an era marked by the rise of targeted therapies and nanomedicine), 2010s, which introduced CRISPR genomics, and the scholarly materials of the 2020 s, marked by the rise of AI algorithms, were relevant to prostate and breast cancer biology. The literature search was performed using Boolean “OR” and “AND” operators between keywords or main terms. The comprehensive literature reviews employing the use of Boolean operators are a well‐established practice that has been used in previous studies [28]. Original and review studies were manually excluded if they were irrelevant to BC and PCa treatment. Further, non‐English articles were also manually excluded (Figure 1). The search result was imported into EndNote Version X8, Clarivate Analytics, USA.

The flowchart methodology used in this review is summarized in Figure 1.

## 3 Prostate Cancer Diagnostic Pathway

### 3.1 General Anatomy of the Prostate Glands

**Figure 2:**
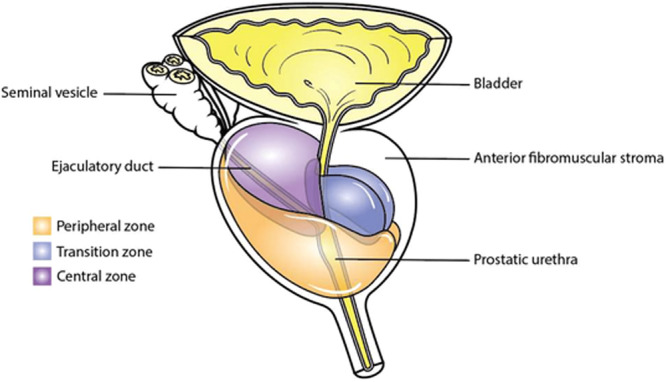
Anatomical zones of the prostate gland [29].

The zonal anatomy of the prostate, as established by McNeal segmentation, consists of three glandular zones: peripheral, transition, and central zones, and the anterior fibromuscular stroma, which is non‐glandular [29, 30]. The central zone lies posterior to the transition zone and surrounds the left and right ejaculatory ducts [31]. The transition zone surrounds the prostatic urethra. The peripheral zone envelops the transition, and the central zone laterally and posteriorly [31]. The anterior fibromuscular is anterior to the transition zone and forms the anterior aspect of the prostate. PCa is commonly found in the periphery zone of the prostate (70%) rather than the anterior fibromuscular stroma zone (5%) and the transition zone (25%) [29], as shown in Figure 2. On the other hand, benign prostatic hyperplasia mainly affects the transition zone. This discrepancy is due to the differences in the densities of glandular tissues susceptible to transformation in each zone. Figure 2 illustrates the zones of the prostate, according to McNeal.

### 3.2 Prostate Cancer Progression

**Figure 3:**
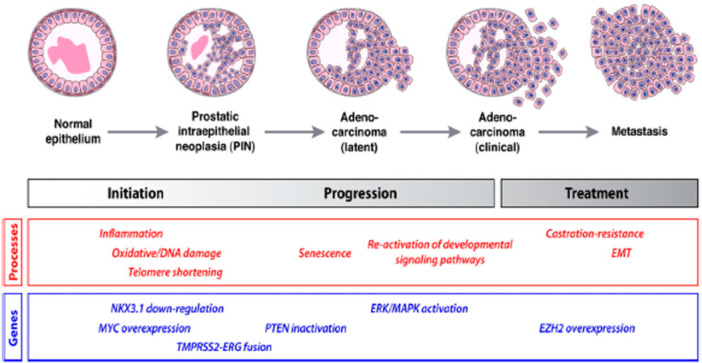
The development of prostate cancer – processes and genes involved [33, 35].

The first sign of PCa is the inflammation of the prostate gland due to rapid and uncontrolled cell division [32]. Clinical observations reveal that the damage to the DNA results in uncontrollable cell division. The oxidative stress resulting from prostate gland inflammation potentially shortens the prostatic telomeres, the onset of PCa. Research has shown that several genes, such as Transmembrane Protease, Serine 2‐ETS‐Related Gene (TMPRSS2‐ERG), NK3 Homeobox 1 (NKX3.1), phosphatase and Tensin (PTEN) Homologue, and myelocytomatosis (MYC) viral oncogene Homologue, are known to initiate PCa [33]. In particular, PMPRSS2‐ERG gene fusion causes the main molecular type of PCa [34]. The reactivation of pathways involved in uncontrollable cell division leads to metastasis of PCa, resulting in rapid cell proliferation (cf. Figure 3).

**Figure 4:**
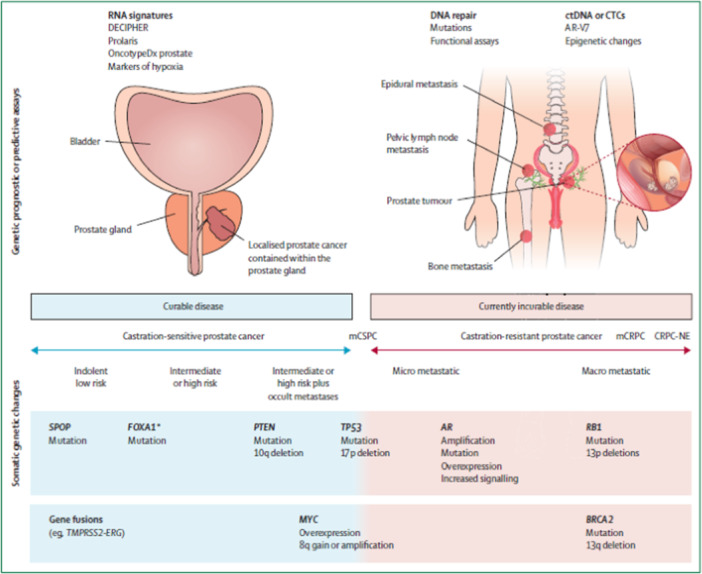
Prostate cancer genetic prognostic or predictive essay and the somatic genetic challenges [38].

The oncogenesis of PCa is linked with complex interactions between acquired somatic gene alterations, inherent germline susceptibility, and micro‐ and macro‐environmental factors [36]. It is believed that chronic infection and inflammation due to urinary microbes drive prostate carcinogenesis through oxidative stress and the generation of reactive oxygen species, which damage the DNA and selection of mutated cells. The proliferative inflammatory atrophy (PIA) identified during biopsies is characterized by shrinkage of the glandular tissue (glandular atrophy), rapidly dividing epithelial cells, and chronic inflammation [32]. PIA is a precancerous lesion but is susceptible to epigenetic and genetic transformation, eventually leading to prostatic intraepithelial neoplasia (PIN) and, finally, lethal cancer cells [32]. PIN can be categorized within the tissue from low‐grade to high‐grade. In high‐grade PIN lesions, the presence of multilayered luminal epithelium is associated with markers of transformation such as cytokeratin 14 (KRT14), cytokeratin 5 (KRT5), cytokeratin 18 (KRT18), basal markers p63 (TP63), and overexpression of methyl acyl‐CoA racemase (AMACR) enzyme, which is associated with malignancy in mucus‐secreting glands (adenocarcinoma) [37, 38]. Figure 4 depicts various prognostic and predictive biomarkers and somatic mutations in localized and metastatic PCa.

In PCa, somatic mutations drive cancer progression and affect how these cancerous cells respond to the tumour. The commonly mutated genes in PCa include RB1, PTEN, and TP53, while ATM and BRCA1/2 are genes involved in DNA repair [33, 39]. Genomic classifiers such as the Gleason score and PSA levels are important prognostic biomarkers that predict the likelihood of cancer progression [32]. Additionally, androgen receptor splice variant 7 (AR‐V7) is a predictive biomarker that predicts tumour resistance to specific hormonal therapies, influencing treatment decisions. TMPRSS2‐ERG is the most common genetic alteration in PCa and prominently contributes to the tumour's initiation and progression. While this fusion is uncommon among men of Asian heritage, in every 4 of 10 prostate tumours, it is a result of hotspot mutations in CHD1, ZNF292, and FOXA1 [33, 38].

### 3.3 Prostate Cancer Diagnosis and Treatment

Conventionally, PCa diagnosis by digital rectal examination (DRE) involves the assessment of the size of the prostate gland by inserting a gloved finger into the patient's rectum [40]. On top of DRE, health screening, magnetic resonance imaging (MRI), prostate‐specific antigen (PSA) testing, and prostate biopsy and analysis have been proposed as PCa screening techniques [41]. Advanced imaging techniques such as multiparametric MRI (mp‐MRI), PCa gene 3 (PCA3) test, which measures PCA3 gene level, and prostate health index (PHI) that combines various forms of PSA [42]. Other additional tests may include the Gleason score to determine the aggressive nature of PCa [43], bone scan to monitor cancer cells in bones [44], and computed tomography (CT) scan to detect PCa accurately [45].

### 3.4 Treatment Options for Localized Prostate Cancer

#### 3.4.1 Radical Prostatectomy

**Figure 5:**
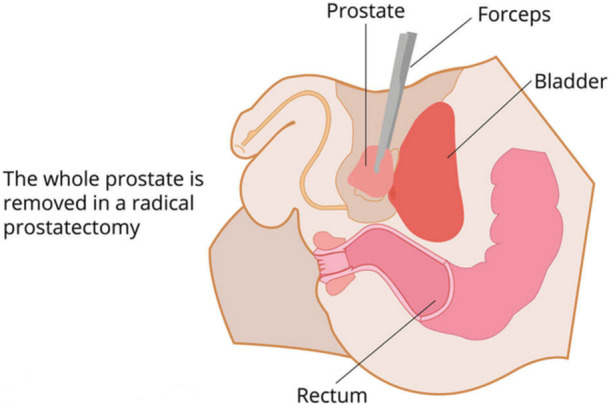
Radical prostatectomy treatment [49].

For healthy men younger than 70 years diagnosed with localized PCa, radical prostatectomy (RP) is recommended [46, 47]. RP involves the removal of the prostate with the prostatic urethra together with the seminal vesicles. RP is performed using a minimally invasive, nerve‐sparing technique with a laparoscopic or robotic‐assisted laparoscopic approach [48]. This can be attributed to the PSA‐based screening; the rate of organ‐confined PCa during diagnosis has increased, leading to successful surgery. Patients with organ‐confined PCa (selected patients with high‐risk disease and low‐ or intermediate‐risk disease), younger than 70 years, with a life expectancy of more than 10 years and minimal or no comorbidities, have benefited from this treatment option [33]. Figure 5 depicts RP whereby the whole prostate is removed to eliminate all the cancerous tissue, and consequently, preserve nerve for erections, minimize tumour recurrence, and prevent spread of tumour cells [49].

**Figure 6:**
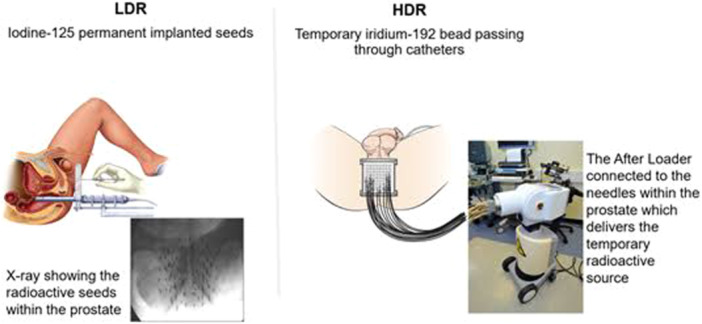
Types of prostate brachytherapy: Low‐dose rate (LDR) and high‐dose‐rate (HDR) [56].

Brachytherapy, also called internal radiation, involves inserting small radioactive sources into the prostate gland using the transperineal approach [50]. This treatment option is the most extreme dose injection, delivered under trans‐ultrasound guidance. This technique takes approximately 45 min to perform under an epidural or general anaesthesia [51]. Brachytherapy may be accomplished by low‐dose rate (LDR), whereby radioactive isotopes such as caesium, palladium, or iodine 125 are permanently placed in the prostate [52, 53]. It may also be delivered through a high‐dose rate (HDR) whereby the catheters are temporarily placed in the prostate while the iridium 192 isotope flows down each catheter, delivering a highly conformal dose over 15 to 20 min [54, 55]. HDR and LDR differ only in the speed of radiation dose delivery. In LDR, the dose is delivered over 12–16 weeks via tiny radioactive seeds implanted permanently on the prostate under short anaesthetic, while in HDR, the dose is given quickly under anaesthetic, typically within 30 min through needles temporarily placed in the prostate [56], as depicted in Figure 6. This treatment option is preferred because of the delivery of high doses of radiation with minimal effect on the adjacent normal tissues due to the rapid dose fall‐off.

A comprehensive study by Shah et al. [25] delved into treatment‐related side effects and cancer‐specific survival outcomes among patients with PCa. In this context, the research group compared the effectiveness of various treatment modalities in managing localized and advanced (metastatic) PCa, along with potential adverse effects as summarized in Table 1.

**Table 1:** Common PCa treatment options and potential adverse effects [25].

Treatment option	Disease progression	Potential adverse effects
Radical prostatectomy	Localized	Urinary incontinence Erectile dysfunction
Active surveillance	Localized	Illness uncertainty
Brachytherapy	Localized	Urinary incontinence Erectile dysfunction Diarrhoea, dysuria, proctitis, urinary urgency, and frequency
External beam radiation	Localized and advanced disease	Urinary incontinence Erectile dysfunction Diarrhoea, dysuria, proctitis, urinary urgency, and frequency
Hormone therapy	Advanced	Hot flashes and flare effect Hyperlipidemia Insulin resistance Cardiovascular disease Anaemia Fatigue Osteoporosis Erectile dysfunction Cognitive deficits
Chemotherapy	Advanced	Peripheral neuropathy Gastrointestinal upset Myelosuppression Hypersensitivity reaction

## 4 Breast Cancer Progression and Treatment

### 4.1 General Anatomy of Breast Cancer

**Figure 7:**
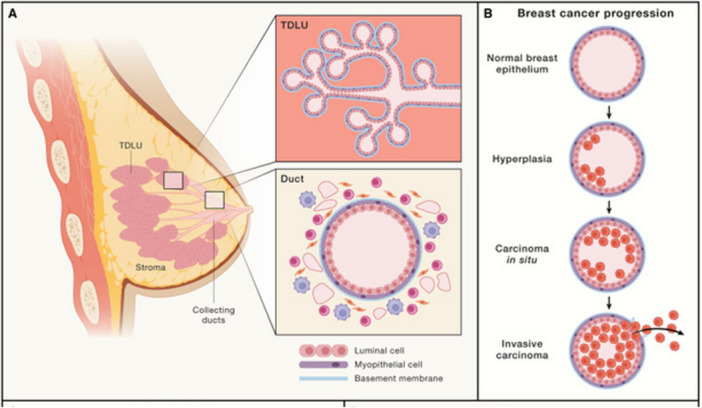
Breast anatomy and histopathological classification of BC (A) and (B) BC progression from normal breast to invasive carcinoma [59].

The breast has various tissues – the lobes and lobules, ducts, nipple and areola, stroma, lymphatic system, and blood vessels. Normal breast tissue has three layers – myoepithelial cells, epithelial cells, and basement membrane [57]. At the onset of BC, the organization of these is interrupted, and the normal tissues fail to function. BC progression starts with hyperplastic lesions, which extend to lobular or ductal in situ, invasive carcinoma, and metastatic cancers [57]. BC manifests in various forms, including changes in breast size and shape, pain in the nipple or breast, thickening of the lumps, nipple discharge, and skin changes (puckering or dimpling) [58]. Figure 7 (A) depicts the anatomy of a normal breast, showing the terminal ductal lobular unit (TDLU), where the majority of the tumours arise, and the breast duct tree, which consists of myoepithelial and luminal cells surrounded by stroma that influences carcinogenesis and physiology of the normal breast [59]. Figure 7B shows BC pathogenesis, cell division, and overgrowth in the ductal or lobular epithelium led to carcinoma in situ (pre‐invasive lesions). Invasive carcinoma is when the tumour cells breach the basement membrane and infiltrate the surrounding stroma [59]. These cancers become metastatic following the degradation of the basement membrane and myoepithelial layer, angiogenesis, stroma cell proliferation, and the invasion of tumorigenic‐epithelial cells to distant sites (see Figure 7). Metastatic cancer cells mainly target sites, including the brain, the lungs, the liver, and the bones [60, 61]. Clinical research has strongly linked metastatic events to an increase in mortality rates among BC patients.

BC is classified molecularly based on histological features and immunohistochemical expression of progesterone receptor (PR), oestrogen receptor (ER), human epidermal growth factor receptor 2 (HER2), and proliferation marker (Ki67) [57]. BC is also clinically stratified into various clinical groups, including triple‐negative breast cancer (TNBC), HER2+ , E+ , and basal‐like [62, 63], as summarized in Table 2. Studies have reported that ER+ tumours account for about 70% of all BCs, where ER+ is defined as greater than or equal to 1% ER‐positive cancer cells [59].

**Table 2:** Clinical stratification of BC based on histological features [59].

Subtype	Characteristics	Prognosis
Luminal A	Estrogen receptor (ER) positive	Has the best prognosis when treated with hormone therapy
	Progesterone receptor (PR) positive	
	Low Ki‐67 index	
	HER2‐negative	
Luminal B	ER‐positive	More aggressive compared to Luminal A
	PR‐positive or PR negative	May require additional treatments such as chemotherapy
	HER2 negative or positive	
HER2‐positive	PR‐negative	More aggressive
	ER‐negative	Treated with targeted therapies, e.g., Herceptin
	HER2‐positive	
Triple‐Negative Breast Cancer (TNBC)	HER2‐negative	Most aggressive
	PR‐negative	Treated with immunotherapy
	ER‐negative	It occurs in younger women
Basal‐like	Overlaps with TNBC but has high basal cytokeratin expression	Most Aggressive
		Has fewer targeted treatment options

### 4.2 Current Deployed in Localized and Systemic BC

In systemic treatment of BC, chemotherapy has been extensively used to suppress the rapid division and proliferation of cancer cells [64]. This method targets cancer cells at different cell cycle stages with the administration of common chemotherapy agents, including cyclophosphamide, platinum agents (carboplatin, cisplatin), taxanes (PTX, docetaxel), and anthracyclines (doxorubicin, epirubicin), among others [65]. There are two types of chemotherapy – neoadjuvant and adjuvant chemotherapy. Neoadjuvant chemotherapy is the chemotherapy applied to patients before the operation [64]. In contrast, adjuvant chemotherapy is chemotherapy delivered after surgery for BC, particularly in cancer tumours that may recur or BC patients with lymphatic metastases [66].

**Figure 8:**
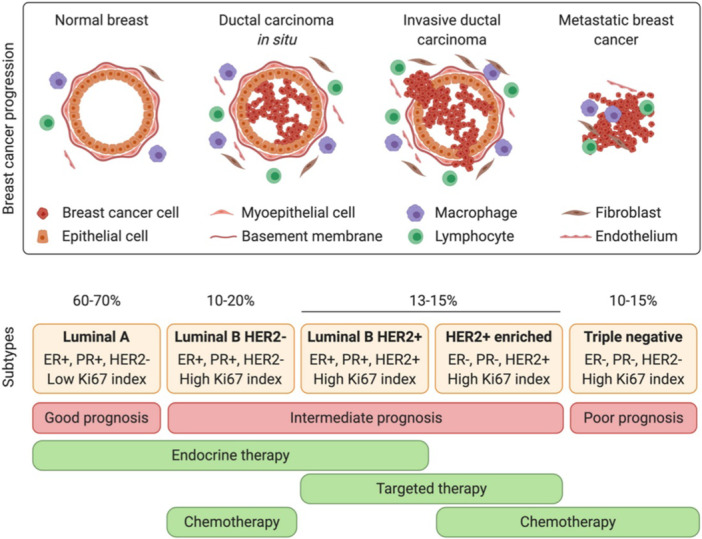
Breast cancer progression from the primary tumour to lethal cancer cells, subtypes of BC, and available pharmacological treatment for each subtype [73].

Immunotherapy is also used in BC treatment, whereby the patient's immunity is strengthened to identify and destroy the tumours precisely [67, 68]. Immunotherapeutic agents have also been shown to help minimize recurrence rates and prevent distant metastasis. Patients with TNBC benefit from this treatment option due to tumour‐infiltrating lymphocytes, mutations, and increased levels of programmed death ligand 1 [69]. HER2 therapy is also a treatment option recommended for patients with HER2‐enriched subtypes [70]. The characteristics of the HER2 subtype are more aggressive development and rapid tumour growth, and have been managed by the use of drugs such as tyrosine kinase inhibitors (lapatinib, neratinib, among others), monoclonal antibodies (pertuzumab and trastuzumab), and antibody‐drug conjugate (trastuzumab emtansine) [71, 72]. Figure 8 presents a summary of BC cancer progression from the primary tumour to lethal cancer cells, subtypes of BC, and available pharmacological treatment for each subtype.

### 4.3 Synergistic Potential of Nanomedicine in Prostate Cancer and Breast Cancer Treatment

Nanomedicine has exhibited great potential in advancing the prevention, treatment, monitoring, and management of biological diseases [74]. According to Yang, He [26], nanotechnology‐based therapies are beneficial in overcoming drug resistance mechanisms, reducing antineoplastic‐related toxicity, prolonging the circulation time of drugs in blood, enhancing solubility and chemical stability of drugs, and delivering drugs to tumour sites via active or passive targeting strategies. Clinically approved nano agents such as Doxil® are currently deployed in ovarian cancer management and various myeloma [75]. Doxil® contains PEGylated liposomal doxorubicin (tiny spherical vesicles) that reduces the effects of the doxorubicin compound on healthy tissues, although it may also cause cardiomyopathy [76]. Doxil® has benefits, including improved tolerability, enhanced drug delivery to the tumour site, and reduced cardiotoxicity [77, 78]. Abraxane® is also another nanotherapeutic drug that the US FDA has approved to manage BC, non‐small‐cell lung cancer, and pancreatic cancer [79, 80]. The albumin‐bound formulations deliver paclitaxel efficiently to tumour sites, reducing the need for solvents to dissolve paclitaxel [81]. The advantage of this drug in cancer treatment is that it is versatile (it can be used to manage pancreatic, BC, and lung cancer [82]. Also, albumin‐bound drugs increase the solubility of the drug and its circulation in the bloodstream. Myocet® is similar to Doxil® ‐ both contain liposomal doxorubicin, which helps to deliver nanodrugs efficiently to the tumours [83].

Myocet® is used in managing metastatic BC and is usually administered intravenously [84, 85]. In some cases, Myocet® can cause hair loss, neutropenia, and thrombocytopenia [85]. Lipusu® is another nano drug used in managing PCa, including castration‐resistant tumours [86]. This drug is known to increase drug bioavailability and reduce multidrug resistance [87]. Furthermore, Nanoxel® is a nanodrug with a paclitaxel formulation. Paclitaxel inhibits cell growth. The drug is administered intravenously every 21 days [88]. Another formulation of paclitaxel is Genexol‐PM®, which uses polymeric micelles to deliver the drug to metastatic BC sites effectively [89, 90]. Another US FDA‐approved nanodrug is Lipodox®, which works similarly to Doxil®. It uses PEGylated liposomes to deliver doxorubicin into the cancer cells effectively [91]. It is also administered every 3 weeks to treat lung, stomach, prostate, and breast cancers [92]. Lastly, Kadcyla® is another approved drug by the FDA that effectively treats HER2+ metastatic BC. The nano drugs that the US FDA has approved for cancer treatment are summarized in Table 3.

**Table 3:** Nanomedicine is used in cancer treatment [73].

Name/Manufacturer	Nanocarrier	Drug/compound	Approval year	Indication	Ref
Doxil® (Janssen Pharmaceutica)	PEGylatedLiposome	Doxorubicin	1995	Metastatic	[93, 94]
Myocet® (Sopherion Therapeutics)	Non‐PEGylatedLiposome	Doxorubicin	2000	Metastatic	[95, 96]
Abraxane® (Celgene)	Albumin	Paclitaxel	2005	Metastatic	[97, 98, 99]
Lipusu® (Sike Pharmaceutical Co. Ltd)	Liposome	Paclitaxel	2006	Non‐metastatic	[100, 101, 102, 103]
Nanoxel® (Fresenius Kabi India Pvt Ltd)	NIPAM‐VP	Paclitaxel	2006	Metastatic	[102, 104]
Genexol‐PM® (Samyang Biopharm)	mPEG‐PDDLA	Paclitaxel	2007	Non‐metastatic	[105, 106]
Lipodox® (Sun Pharma Global FZE)	PEGylatedLiposome	Doxorubicin	2013	Metastatic	[107, 108]
Kadcyla® (Hoffmann‐La Roche	Antibody	Trastuzumab/DM1	2013 2019	Metastatic HER2+, Residual disease	[109, 110, 111]

### 4.4 Clinical Applications of Artificial Intelligence in the Cancer Genome Atlas

With the emergence of artificial intelligence (AI), AI‐optimized drug nanomedicine combinations have surpassed randomly chosen drug combinations. Patients with metastatic castration‐resistant PCa have benefited from an AI‐driven platform (CURATE AI) that refined treatment regimens, supporting evidence of CURATE AI in addressing big data challenges in personalized treatment and providing information on the safety and efficacy of lead candidates in oncology trials. AI synergy with nanomedicine is instrumental in forecasting tumour accumulation, side effects, and biodistribution of nanoparticles. AI is already a game‐changer in cancer research, especially in imaging and genomics. Machine learning models detect patterns in radiologic images, allowing earlier diagnosis and more accurate staging [112]. Genomic deep learning models can detect biomarkers and predict therapeutic response more precisely [113]. AI has been applied in prostate and breast cancer to enhance risk stratification, treatment regimens, and recurrence prediction. The Cancer Genome Atlas (TCGA) has established itself as a pillar of oncology, offering multi‐omics data on many cancer types. These datasets support pan‐cancer multi‐gene profiling to single‐gene analysis [114].

The volume of TCGA data requires sophisticated analytical solutions, and AI has turned out to be an invaluable component [115]. Limitations of bulk transcriptomic repositories should be considered to preserve balanced interpretation. AI, such as machine learning classifiers, deep learning pipelines, and integrative bioinformatics pipelines, complements TCGA analyses to identify biomarkers, forecast prognosis, and therapeutic targets. Unlike standard statistics, AI captures nonlinear dependencies, heterogeneous data types, and scales to pan‐cancer analysis. Specific gene research in one cancer is still pioneering. According to Liu and Weng [116], CDK2 expression correlated with glioma progression. Li and Liu [117], as well as Liu and Li [118], emphasize gene‐specific prognostic potential. These studies can be automated with AI to refine feature selection, find unobservable predictors of survival, and visualize pathways using deep learning models that outperform regression. Convolutional neural networks might combine histopathological images and gene expression, providing multimodal evidence reinforcing single‐gene analyses. Single‐gene pan‐cancer analyses exemplify how biomarkers cross tumour borders [119, 120, 121]. AI enhances these studies with clustering algorithms and transfer learning, extrapolating biomarker applicability to cancer types. This cross‐cancer lens is useful in defining universal therapeutic targets and stratifying patients in precision oncology. Latent biomarker potential exists, as deep learning models detect subtle expression patterns missed by conservative analyses.

Investigations like Liu and Tang [119], Liu [122], and Liu et al. [123] unveil how clusters of genes categorize patients. AI excels here, employing multi‐gene signatures in prediction models, nonlinear interactions, and risk scores that outperform traditional analytics. Ensemble learning schemes integrate gene signatures into complex predictors, and autoencoders shrink features without eliminating biological content. Multi‐gene analysis over pan‐cancer is where AI is most scalable. Literature showcases how AI calculates multidimensional gene networks, combining transcriptomic and epigenomic data to discover pathways applicable to cancers [122, 124, 125, 126]. Deep learning architectures provide insights into latent structure within high‐dimensional data, aiding biomarker and therapeutic target development. Graph neural networks can model gene‐gene interactions across cancers and reveal conserved oncogenic signatures. Results can be affected by sample composition biases, including over‐representation of demographics or under‐representation of subtypes. Technical bias, such as batch effects and sequencing variance, complicates interpretation [127, 128]. AI can manage them with domain adaptation, adversarial learning, and batch effect correction. Underrepresented subtypes can be balanced with transfer learning. However, AI is not a panacea; biases in inputs permeate models. Transparency, cross‐cohort validation, and prudent interpretation are vital. Training models based on TCGA data must be evaluated on external datasets, e.g., METABRIC or GEO, to validate generalization. Single‐cell data could circumvent bulk transcriptomics limitations by addressing cellular heterogeneity [123].

### 4.5 Clinical Application of Artificial Intelligence in Breast Cancer and Prostate Cancer Management

**Figure 9:**
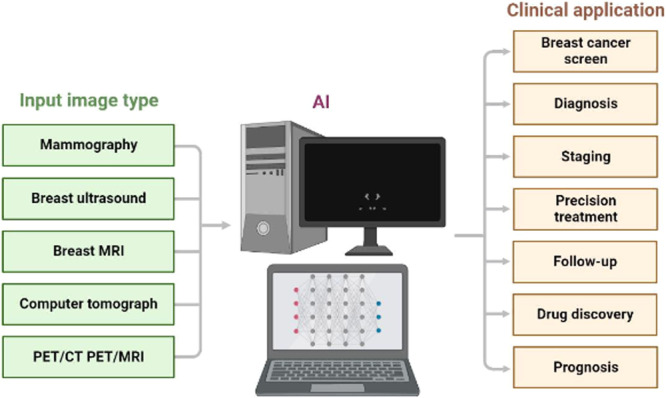
Clinical Application of artificial intelligence in breast cancer diagnosis [130].

Artificial intelligence (AI) is broadly classified into deep learning (DL) and traditional machine learning (ML) [129]. The former relies on automatically extracted features by convolutional neural networks (CNN), enabling the detection of complex information that is difficult for the human brain, while the latter utilizes hand‐crafted features such as texture and colour [129]. Recent advances in AI‐assisted imaging have shown huge potential in automatically diagnosing, recognizing, and segmenting suspicious tumour lesions [130, 131]. AI has immense potential in biomarker evaluation, staging, and prognostication [132]. AI has enormous prospects in performing tasks beyond grading and diagnosis, but it can also be used in measuring cell volume and length, quantification of cribriform, quantification of immunohistochemistry, and recognition and quantification of perineural invasion [132]. BC patients have benefited from AI‐assisted imaging diagnosis in various ways: early detection of tumours through precise analysis of the mammograms, minimizing unnecessary biopsies (often associated with false positives), helping in the analysis of patients' genetic information, which guides the development of personalized treatment plans, and AI‐powered software is highly efficient in the interpretation of breast imaging [132]. In PCa contexts, AI has been used to distinguish between malignant and benign tumours, identify molecular subtypes, grade Gleason, and predict clinical prognosis [133]. Accordingly, AI models have helped urologists make clinical decisions and stratify patient risks. Figure 9 shows the clinical application of BC diagnosis and treatment. However, AI systems require clinical validation to be effective in clinical settings [134]. Another scientific challenge is also associated with generalizing the AI models across different settings and demographic groups. It is well‐established that different AI models, such as deep algorithms, operate as “black boxes”, impeding accurate explanations of the findings [135].

## 5 Conclusions and Future Outlook

The evolution of molecular biology and the application of combinatorial chemistry, structural biology, and computer‐aided drug design have benefited anti‐cancer drug development. Both PCa and BC incidences are increasing yearly and demanding more attention. Drug resistance has emerged as a considerable obstacle to optimized treatments, attributed to enhanced DNA repair of tumour cells, defects in apoptosis, target changes, and enhanced efflux mechanisms. Despite advances, further research is necessary to overcome these challenges. Next‐generation technologies should improve diagnostic pathways, minimize recurrence, and optimize treatment deliveries. Therapeutic studies should focus more on tumour biology and early biomarker detection to develop individualized treatment plans. Advances in computational chemistry have played a key role in polypharmacology, leading to agents acting on multiple pathways or targets. Nanotechnologies have been designed to deliver chemotherapy drugs, radionuclides, and hormones to tumour sites. This technology has also been combined with conventional treatments such as hormone therapy, radiation, and surgery to deliver drugs more precisely and spare healthy cells.

The future of BC and PCa treatment is poised to transform, enhancing the specificity and sensitivity of diagnostic tools. Nanoparticles have been used to detect PSA and other biomarkers. Current research will transition toward the convergence of AI and nanomedicine. Adaptive regulatory pathways should allow iterative improvements depending on in vivo and in vitro analysis, bridging the gap between lead identification and implementation. Pathologists should not rely only on AI prediction but also on diagnostic skills. Collaboration between AI experts, regulatory bodies, and pathologists can integrate AI and nanomedicine into clinical systems. Future research should prioritize robust AI systems and integrate generative AI tools such as ChatGPT. Nanotechnology will guide precision medicine targeting molecular markers or gene mutations, ensuring less toxic, more effective treatments and reduced resistance. Nanotheranostics offer opportunities to enhance diagnostic accuracy, minimize side effects, and enable targeted drug delivery. AI integration has improved early detection, monitored treatment response, and predicted recurrence.

## Author Contributions

**Russell B.O. Ouma:** method development, writing and editing; **Silas M. Ngari:** visualization, editing, and supervision. All authors have read and approved the manuscript. All authors have read and approved the final version of the manuscript. Joshua K. Kibet had full access to all of the data in this study and takes complete responsibility for the integrity of the data and the accuracy of the data analysis; **Joshua K. Kibet:** conceptualization, editing and supervision.

## Funding

The authors received no specific funding for this work.

## Ethics Statement

The authors have nothing to report.

## Consent for Publication

This article has the consent of all the authors.

## Conflicts of Interest

The authors declare no conflicts of interest.

## 1 Transparency Statement

The lead author Joshua K. Kibet affirms that this manuscript is an honest, accurate, and transparent account of the study being reported; that no important aspects of the study have been omitted; and that any discrepancies from the study as planned (and, if relevant, registered) have been explained.

## Data Availability

The data associated with the findings of this study are available from the corresponding author upon reasonable request.
